# A Review of On-Surface Synthesis and Characterization of Macrocycles

**DOI:** 10.3390/nano15151184

**Published:** 2025-08-01

**Authors:** Chao Yan, Yiwen Wang, Jiahui Li, Xiaorui Chen, Xin Zhang, Jianzhi Gao, Minghu Pan

**Affiliations:** 1 School of Physics and Information Technology, Shaanxi Normal University, Xi’an 710119, China; chaoyan@snnu.edu.cn (C.Y.); 2023300886@snnu.edu.cn (Y.W.); lijiahui1008-@snnu.edu.cn (J.L.); minghupan@hust.edu.cn (M.P.); 2 School of Mathematics and Physics, Xinjiang Agricultural University, Urumqi 830052, China; 3 School of Mechanical and Material Engineering, Xi’an University, Xi’an 710065, China; xrchen@xawl.edu.cn

**Keywords:** on-surface synthesis, organometallic or covalent macrocycles, Ullmann coupling, scanning probe microscopy

## Abstract

Macrocyclic organic nanostructures have emerged as crucial components of functional supramolecular materials owing to their unique structural and chemical features, such as their distinctive “infinite” cyclic topology and tunable topology-dependent properties, attracting significant recent attention. However, the controlled synthesis of macrocyclic compounds with well-defined compositions and geometries remains a formidable challenge. On-surface synthesis, capable of constructing nanostructures with atomic precision on various substrates, has become a frontier technique for exploring novel macrocyclic architectures. This review summarizes the recent advances in the on-surface synthesis of macrocycles. It focuses on analyzing the synthetic mechanisms and conformational characterization of macrocycles formed through diverse bonding interactions, including both covalent and non-covalent linkages. This review elucidates the intricate interplay between the thermodynamic and kinetic factors governing macrocyclic structure formation across these bonding types and clarifies the critical influence of the reaction temperature and external conditions on the cyclization efficiency. Ultimately, this study offers design strategies for the precise on-surface synthesis of larger and more flexible macrocyclic compounds.

## 1. Introduction

Macrocycles refer to a class of molecular structures characterized by a cyclic backbone with a large ring size (typically containing more than 12 atoms), where the internal cavity diameter generally falls within the nanoscale. These molecules have attracted significant interest because of their unique cavity structures, tunable π-conjugated systems, and surface-confined reaction characteristics. Their conformational flexibility makes them important components of functional supramolecular materials. Compared to open-chain oligomers, cyclic topologies confer distinct physicochemical and optoelectronic properties, such as higher density, lower intrinsic viscosity, and enhanced thermal stability [1,2,3]; spin collective excitation [4,5]; and amplified nonlinear optical responses [6]. Macrocycles with extended conjugation have potential applications in organic solar cells, organic electronics, and drug discovery [7,8,9].

On-surface synthesis is an effective method for fabricating organic nanostructures with atomic precision [10,11,12]. Notably, the on-surface Ullmann coupling (a method for achieving covalent intermolecular coupling on solid surfaces via metal catalysis) enables atomic-scale precision control over reaction pathways and product-rich supramolecular structures [13,14]. It allows for the construction of large-scale conjugated molecular systems on substrates and facilitates real-time observation of reaction intermediates and final products using scanning tunneling microscopy (STM), thereby deepening the understanding of reaction mechanisms. This method holds significant importance in nanomaterial fabrication and molecular device construction; the detailed reaction principles and product characteristics will be discussed further in subsequent experimental case studies. However, on-surface synthesis often involves irreversible covalent coupling, precluding self-repair processes during the reaction, and consequently, defects become permanent [15,16,17]. Therefore, the design of precursor molecules is crucial for triggering selective coupling or controlling reaction kinetics to generate target nanostructures. To date, strategies for synthesizing macrocycles on surfaces remain limited and are primarily achieved through pseudo-high dilution, template effects, and atomic manipulation [18,19,20,21]. The synthesis of giant macrocycles poses considerable challenges, as it demands the precise organization of a large number of precursor monomers. Furthermore, cyclization reactions compete with chain extension reactions.

**Scheme 1:**
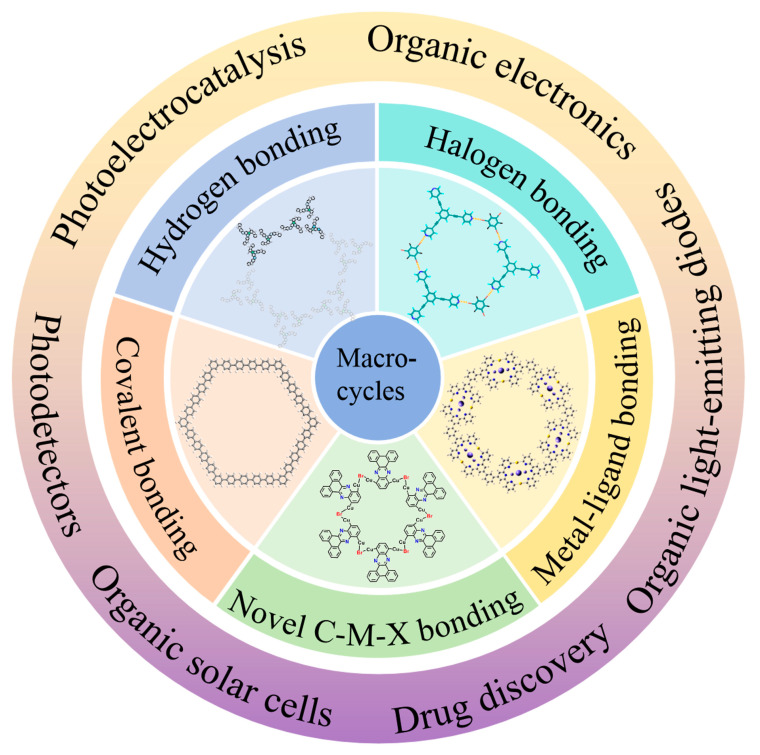
Distinct bonding types and applications in macrocycles via surface synthesis [26,27,28,29,30].

Advances in scanning probe microscopy (SPM), particularly ultra-high vacuum scanning tunneling microscopy (UHV-STM) and non-contact atomic force microscopy (nc-AFM), now enable the “visualization” and detailed structural and electronic characterization of intermediates and products in surface reactions at the single-molecule level [14,21,22,23,24,25]. Additionally, combined with density functional theory (DFT) calculations and molecular dynamics (MD) simulations, these techniques further validate the product structures and the elucidate reaction mechanisms. This review surveys recent studies on on-surface-synthesized macrocycles, with a specific focus on 2D structures on the surface (Scheme 1).

It focuses on analyzing the conformational characterization and cyclization mechanisms under different chemical bonds, such as hydrogen, halogen, metal–ligand, C–M–X, and covalent bonds. The influence of external environmental variables, including substrate selection, annealing parameters, molecular coverage, and external electric fields, on product structures was also explored. This analysis provides a foundation for developing relatively simple methods for preparing high-yield, shape-controllable, and large-sized macrocycles.

## 2. Metal–Ligand Bonding

On-surface synthesized macrocycles have attracted significant interest due to their rich and diverse topological structures [31]. However, synthesizing macrocycles with well-defined compositions and geometries via simple methods remains a daunting challenge, particularly for large-sized rings.

**Figure 1:**
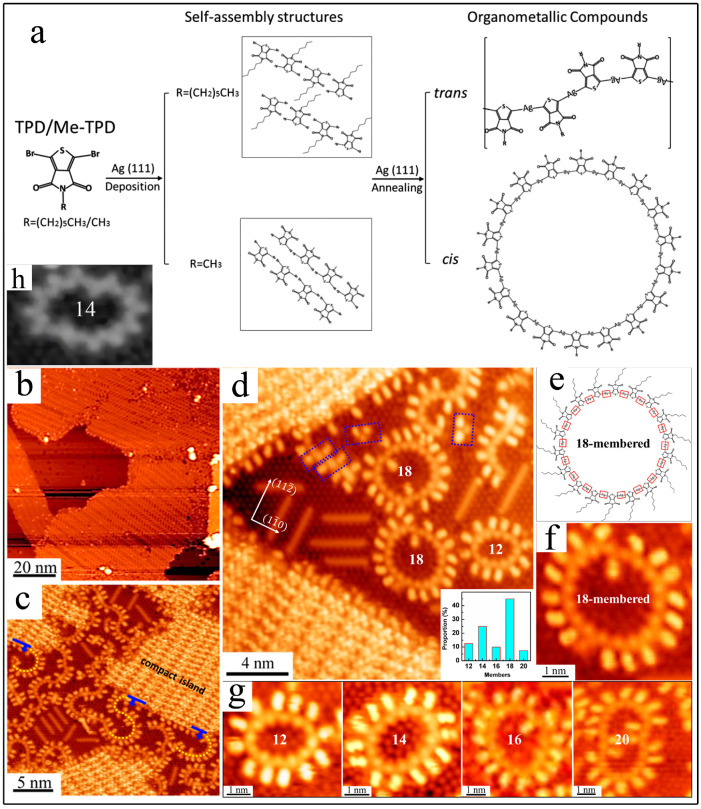
(**a**) Main reaction pathways of TPD and Me–TPD molecules on the Ag(111) substrate. (**b**) Large-scale STM image of TPD molecules on Ag(111) at RT. (**c**) Large-scaled STM image obtained after annealing the sample at 120 °C for 10 min (I = 84 pA, V = 2 V). (**d**) STM image containing three organometallic rings. The inset shows the proportion of various types of organometallic rings (I = 57 pA, V = 2 V). (**e**) Scheme of the 18-membered ring. (**f**) High-resolution STM image of the 18-membered macrocycle (I = 57 pA, V = 2 V). (**g**) STM images of other organometallic structures of 12-membered, 14-membered, 16-membered, and 20-membered rings. (**h**) STM image of 14-membered ring composed of Me–TPD molecules (4.28 × 2.99 nm^2^) [32].

Zhang et al. [32] successfully synthesized large organometallic macrocycles on an Ag(111) surface using 1,3-dibromo-5-hexyl-4H-thieno[3,4-c]pyrrole-4,6(5H)-dione (TPD) as the precursor. The macrocyclic structures were investigated using STM combined with DFT. Figure 1a illustrates the reaction pathways of TPD and Me–TPD precursor molecules on the Ag(111) surface. External alkyl chains of varying lengths are denoted by the letter R. At room temperature (RT), these molecules self-assemble into an ordered structure. After annealing, the bromine atoms dissociated, leading to the formation of metal–organic coordination products with C–Ag–C bonds. Consequently, organometallic (TPD–Ag)_n_-linked cis-cyclic and trans-chain configurations were produced. The ideal macrocycle consists of 18 (TPD–Ag) segments, although rings with other numbers of units were also observed. The self-assembled structure of TPD deposited on the Ag(111) surface is shown in Figure 1b, exhibiting an ordered chain-like structure at RT. After annealing at 120 °C for 10 min, the structure underwent significant changes, resulting in dense islands as well as cis-cyclic and trans-chain configurations (Figure 1c). The macrocycles with 12, 14, 16, 18, and 20 units (Figure 1d,g) appeared when the coordinating molecules connected in a cis configuration. The internal diameters of these rings ranged from 2.62 ± 0.02 nm to 4.22 ± 0.02 nm. The interior of each macrocycle consists of equally spaced dots corresponding to TPD molecules’ backbones, while the outer ring comprises a regular array of short rods representing the long alkyl chain tails. The inset in Figure 1d shows the 18-unit macrocycle as the predominant product. This is attributed to the internal angle of 160° between the two C–Br bonds on the thiophene unit. The ideal macrocycle, depicted in Figure 1e, should consist of 18 molecules in a linear C–Ag–C configuration. However, the experiments revealed that in some 18-unit rings, as well as in 16- and 20-unit rings, a few alkyl chains projected inward (Figure 1f,g), which was attributed to repulsive interactions between the termini of adjacent alkyl chains.

Subsequently, the authors also selected Me–TPD for investigation, following room-temperature deposition of Me–TPD molecules on Ag(111), where annealing the sample at 120 °C for 10 min also led to the formation of organometallic rings (Figure 1h), further demonstrating the feasibility of this approach. The deliberate modulation of alkyl chain length and peripheral functional group identity on thiophene-based precursors emerges as a critical strategy for expanding the structural and functional diversity of organometallic macrocycles. This synthetic control is essential for systematically tailoring their physicochemical properties and ultimately identifying candidates optimized for targeted applications. More importantly, this synthesis strategy, which cleverly exploits the internal angle of organic molecules and surface reactions, provides a practical method for synthesizing larger and more flexible organometallic macrocycles.

**Figure 2:**
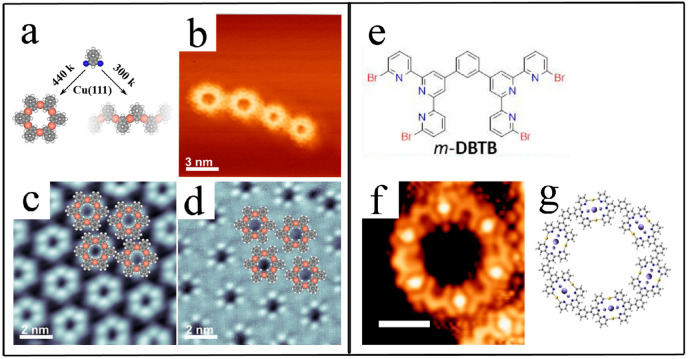
(**a**) Space-filling model showing the possible cyclic (left) and open-chain (right) organometallic reaction products of 1,3-dibromoazulene (DBAz) on Cu(111). Color code: white—hydrogen; grey—carbon; red—copper; blue—bromine. (**b**) STM image of two octamers (left) and two hexamers (right) after annealing to 470 k, with the azulene building blocks visible. U = −0.37 V, I = −0.73 nA. STM images of an island of the cyclic hexamer macrocycles after annealing 470 k for 5 min, taken at different tunneling conditions, with overlaid molecular models: (**c**) V = −0.94 V, I = −0.10 nA and (**d**) V = 1.79 V, I = 0.10 nA [33]. (**e**) Chemical structure of 1,3-bis(6,6″-dibromo-[2,2′:6′,2″-terpyridin]-4′-yl)benzene (m-DBTB). (**f**) The C–Au–C organometallic units after annealing to 457 K; scale bars: 2 nm, V = −1.0 V, I = 1.0 nA. (**g**) Corresponding structural models [26].

Krug et al. [33] investigated organometallic oligomers formed from 1,3-dibromoazulene (DBAz) on Cu(111). Following room-temperature deposition and subsequent annealing at 440 K for 5 min, cyclic organometallic hexamers, as shown in Figure 2a (left), were formed. High-resolution STM imaging under distinct tunneling conditions (Figure 2c,d) provides conclusive evidence for the precise spatial arrangement within the hexameric assemblies. Critically, the topographic prominence of the copper atoms under specific conditions (Figure 2c) allows for unambiguous identification of the organometallic coordination nodes. Furthermore, imaging parameters optimized for molecular orbitals reveal the characteristic bright and uniform features associated with the azulene units (Figure 2d), thereby directly visualizing the integration and electronic signature of these organic components within the macrocyclic framework. Upon further annealing to 470 K, a small number of octameric macrocycles were observed (the two macrocycles on the left in Figure 2b). Xing et al. [26] co-deposited m-DBTB molecules (Figure 2e) with Fe atoms on an Au(111) surface at 293 K. Subsequent annealing at 457 K induced a debrominative coupling reaction, leading to the formation of C–Au–C organometallic macrocycles composed of six m-DBTB molecules (Figure 2f). These macrocycles exhibit a diameter of approximately 3 nm, with Figure 2g showing a structural model of the organometallic macrocycle.

Within this context, Krug et al. also explored the ring/chain competition in surface oligomerization reactions. This phenomenon was explained based on kinetic and thermodynamic principles: At room temperature, kinetic control under non-equilibrium conditions favors entropy-driven chain formation, while at high temperatures, thermodynamic equilibrium shifts the preference toward enthalpy-driven rings. On the other hand, the ring-to-chain ratio exhibits coverage dependence, decreasing with higher coverage as the second-order chain growth outcompetes first-order ring closure.

## 3. Covalent Bonding

### 3.1. C–C Coupling

Conjugated macrocycles refer to cyclic structures that themselves contain an extended, delocalized π-electron system. These rings are typically large in size (usually 1–2 nanometers or larger) [34]. The backbone of the ring consists of continuous conjugated units (such as benzene rings, pyrrole, thiophene, acetylene bonds, etc.) connected together, enabling effective π-electron delocalization around the entire inner perimeter (and sometimes the outer perimeter) of the ring. This forms a unified, cyclic π-electron cloud [35,36,37,38]. This delocalization often imparts unique aromaticity or antiaromaticity to the molecule, along with exceptional photophysical, photochemical, and electrochemical characteristics. Consequently, conjugated macrocycles have attracted significant attention due to their potential applications in areas such as organic solar cells, photodetectors, drug discovery, and organic electronics [39,40,41,42,43].

**Figure 3:**
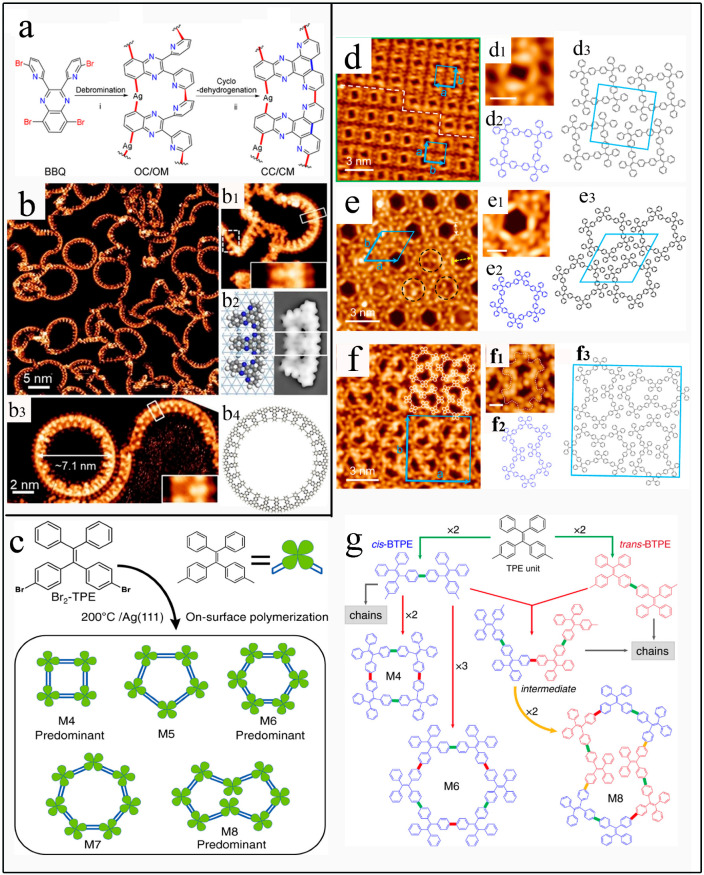
(**a**) Scheme of giant macrocycles synthesized on Ag(111). (**b**) STM overview of CCs and CMs after annealing the sample at 523–594 K. (**b_1_**) High-resolution image of a CC (V = −0.8 V, I = 0.3 nA). (**b_2_**) DFT-optimized adsorption geometry and simulated STM image of an analog of the DPP trimer (V = −1.0 V). (**b_3_**) A typical CM containing 26 subunits (V = −1.0 V, I = 0.3 nA). (**b_4_**) Tentative structural model of (26) DPP [44]. (**c**) On-surface synthesis of tetraphenylethylene macrocycles. (**d**) Zoomed in STM image (2.0 V, 200 pA) of the M4 2D crystal with a domain boundary (dashed line). (**d_1_**) High-resolution STM image (−1.0 V, 150 pA) and (**d_2_**) chemical model of M4. Scale bar: 1 nm. (**d_3_**) Schematic model of the M4 2D crystal. (**e**) Zoomed in STM image (−1.0 V, 100 pA) of the M6 2D crystal. (**e_1_**) High-resolution STM image (0.2 V, 100 pA) and (**e_2_**) chemical model of M6. Scale bar: 1 nm. (**e_3_**) Schematic model of the M6 2D crystal. (**f**) Zoomed in STM image (−1.0 V, 50 pA) of the M8 2D crystal with overlaid simplified molecular models. (**f_1_**) High-resolution STM image (−1.0 V, 150 pA) and (**f_2_**) chemical model of M8. Scale bar: 1 nm. (**f_3_**) Schematic model of the M8 2D crystal. (**g**) Proposed reaction pathways. Multi-step reaction pathways for the formation of M4, M6, and M8 macrocycles [45].

Fan et al. [44] utilized 5,8-dibromo-2,3-bis(6-bromopyridin-2-yl)quinoxaline (BBQ) as the precursor molecule to synthesize a π-conjugated macrocycle with a diameter of 7 nm on Ag(111) via on-surface synthesis. Figure 3a outlines a sophisticated stepwise thermal annealing strategy pivotal for achieving controlled covalent and organometallic assembly. Initial debromination within a lower temperature window (427–499 K) serves as the critical activation step, enabling the interconnection of molecular building blocks through a combination of C–C covalent bonds and C–Ag organometallic linkages (step i). Subsequent elevation of the thermal energy (523–594 K) drives the essential cyclodehydrogenation process (step ii). This concerted intramolecular fusion not only planarizes the structure but crucially generates the desired extended π-systems: arched conjugated chains (CCs) and conjugated macrocycles (CMs). Figure 3b shows STM images of the arched chains and macrocycle, with Figure 3(b_1_,b_3_) presenting higher-resolution views. The high-resolution images revealed that the outer edges of the CCs and CMs exhibited a planar and continuous circular appearance, while the inner edges displayed discontinuous dot-like features. The white dashed box in b1 highlights a T-shaped byproduct formed through the intramolecular cyclodehydrogenation of BBQ, resulting in a dipyridophenazine (DPP) subunit. The deliberate parallel alignment of DPP subunits, achieved through thermally activated debrominative C–C coupling between adjacent pyridine (Py) molecules, serves as a key design strategy for constructing well-defined conjugated chains (CCs) and macrocycles (CMs). DFT simulations based on a DPP trimer (Figure 3(b_2_)) yielded features consistent with the experimental observations. Figure 3(b_3_) shows a macrocycle with a diameter of 7.1 nm, containing 26 DPP subunits, designated as (26) DPP. The structural model of the macrocycle is given in Figure 3(b_4_). Their findings provide a novel design strategy for the precise synthesis of π-conjugated macrocycles and unlock new opportunities for the precise synthesis of organic nanostructures.

Li et al. [45] synthesized conjugated tetraphenylethylene (TPE) macrocycles using 4,4′-(2,2-diphenylethene-1,1-diyl)bis(bromobenzene) (Br_2_-TPE) as the precursor molecule. Employing a pseudo-high-dilution strategy, they utilized surface coupling reactions to achieve this synthesis. Figure 3c presents the macrocyclic products formed by depositing Br_2_-TPE on Ag(111) at 200 °C, where C–Br cleavage generated macrocycles (M4–M8). Even-membered rings (M4/M6/M8) predominated over odd-numbered ones (M5/M7) and spontaneously organized into mono-component 2D crystals. This highly efficient self-classification behavior underscores the exceptional structural precision achievable through this approach and highlights its potential for generating complex supramolecular architectures.

Figure 3d depicts the 2D crystal composed of M4 macrocycles. The unit cell is not strictly square but slightly elongated in one direction, with lattice parameters a = 2.01 ± 0.02 nm, b = 2.06 ± 0.03 nm, and θ = 90 ± 1°, and the unit cells rotate by 90° across the dashed line. Figure 3(d_1_,d_2_) show the STM image and chemical model of the M4 macrocycle, respectively. The four corners of the M4 macrocycle consist of four X-shaped TPE units. Figure 3(d_3_) provides a model of the 2D crystal unit cell. Figure 3e displays the 2D crystal composed of M6 macrocycles. The rhombic unit cell has parameters a = b = 2.85 ± 0.02 nm and θ = 60 ± 1°. The cavity diameter of the M6 macrocycle is 1.69 ± 0.03 nm, which is sufficiently large to accommodate a TPE molecule. Figure 3(e_1_,e_2_) present the STM image and chemical model of the M6 macrocycle, respectively. The six corners of the M6 macrocycle were formed by six X-shaped TPE units. Figure 3(e_3_) shows the unit cell model of the 2D crystal. The M6 macrocycles possess tilted outer phenyl rings that approach each other in a tilted T-shaped configuration, potentially giving rise to weak π–π interactions [46]. Since each M6 macrocycle has 12 phenyl rings participating in π–π interactions, it is inferred that the collective π–π interactions stabilize the 2D crystalline phase. Figure 3f illustrates the 2D crystal composed of M8 macrocycles. The M8 macrocycle adopts an oval-like Cassini shape. Unlike the convex polygons of the M4 and M6 macrocycles, the M8 macrocycle exhibits a concave form. A rectangular unit cell (a = b = 5.92 ± 0.08 nm) is shown in Figure 3f. The analysis of Figure 3(f_1_,f_2_) reveals that the unit cell comprises four M8 macrocycles, with adjacent macrocycles rotated by 90° relative to each other, forming a densely packed basket-like structure. Figure 3(f_3_) provides a schematic of the unit cell, illustrating the interaction of adjacent M8 macrocycles via π–π interactions between their outer phenyl rings.

Figure 3g shows the reaction pathway for the formation of M4, M6, and M8 macrocycles, revealing a multi-step cyclization mechanism. Initially, two TPE monomers underwent debrominative coupling to form cis- or trans-dimers (BTPE). cis-BTPE coupling leads to the formation of M4 and M6 macrocycles, while cross-coupling between cis-BTPE and trans-BTPE forms the M8 macrocycle. Their findings provide a design strategy for the precise on-surface synthesis of multicomponent macrocycles. This research paves new avenues for exploring the photophysical properties of TPE macrocycles.

Since the pioneering work of Ullmann et al. [47], Ullmann coupling has been widely employed in on-surface synthesis. This is a classical method in solution chemistry for preparing complex aryl derivatives [48], favored for its tunable reaction conditions and pre-designed reaction pathways [49]. Ullmann-type coupling reactions on surfaces can be initiated by activating molecular precursors through thermal annealing [50], SPM tip manipulation [51], or light irradiation [52]. The reaction typically involves the following steps: dehalogenation of precursor molecules on the surface to form stable radicals, diffusion of these radicals across the surface, and their subsequent engagement in one of two potential reaction pathways, depending on the surface properties. The first pathway involves direct radical polymerization, leading to covalent bond formation. The second pathway involves the combination of radicals with metal adatoms to form organometallic (OM) intermediates, followed by the removal of the adsorbed metal atoms upon further annealing to yield covalent structures [53,54].

**Figure 4:**
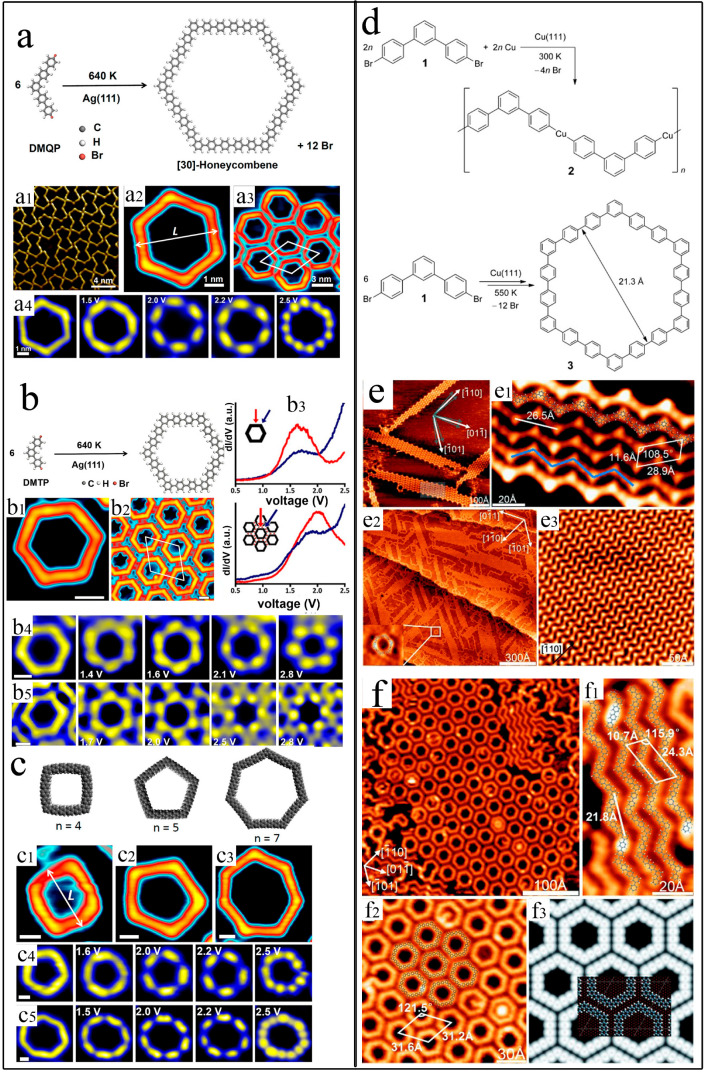
(**a**) Formation of the cyclotriacontaphenylene macrocycle (30)-honeycombene (HC) from DMQP. (**a_1_**) STM image of DMQP on Ag(111) adsorbed at 260 K (78 K imaging, 0.01 V, 3 nA); (**a_2_**) single (30)-HC macrocycle after heating to 640 K (4.4 K, 0.1 V, 0.02 nA); (**a_3_**) (30)-HC island (78 K, 0.2 V, 0.4 nA); (**a_4_**) electronic structure of (30)-HC: topographic image (left, 0.1 V, 0.5 nA) and dI/dV maps (4.4 K, 1.5–2.5 V). (**b**) Formation of the (18)-HC from DBMT 4,4″-dibromo-meta-terphenyl (DBMT). (**b_1_**) STM image of an isolated (18)-HC macrocycle on Ag(111), (4.4 K, −0.03 V, −0.1 nA); (**b_2_**) (18)-HC island (4.4 K, 0.01 V, 0.02 nA); (**b_3_**) dI/dV spectra for (18)-HC; (**b_4_**) topographic image (left) and dI/dV maps (4.4 K, 1.4–2.8 V); (**b_5_**) as above, but for a macrocycle in an island. Scale bar: 1 nm. (**c**) Strained macrocycles formed as side products by on-surface reaction of DMQP as calculated using DFT. STM image of the (**c_1_**) square (20)-HC (0.1 V, 0.02 nA), (**c_2_**) pentagonal (25)-HC (0.05 V, 0.02 nA), and (**c_3_**) heptagonal (35)-HC (0.1 V, 0.02 nA). (**c_4_**) Electronic structure of the (25)-HC: topographic image (left, 1.4 V, 1 nA) and dI/dV maps (1.6–2.5 V); (**c_5_**) (35)-HC: topographic image (left, 1.4 V) and dI/dV maps (1.6–2.5 V). Scale bar: 1 nm [27]. (**d**) The scheme of Ullmann coupling reaction of DMTP 1 forming coordination polymer 2 at 300 K and hyperbenzene molecule 3 at 550 K on Cu(111). (**e**) STM image after deposition of DMTP 1 onto Cu(111) at 300 K at low coverage (−3.6 V, 0.02 nA). (**e_1_**) Higher-resolution image of the shaded area in (**e**) (−3.6 V, 0.01 nA). (**e_2_**) The coordination polymer 2 at higher precursor coverage (−2.75 V, 0.08 nA). (**e_3_**) After deposition of 1 onto Cu(111) held at 440 K (−3.6 V, 0.02 nA). (**f**) STM image after deposition of DMTP 1 onto Cu(111) held at 550 K. (**f_1_**) Magnified view of a small section with oligophenylene chains. (**f_2_**) Magnified view of a hyperbenzene 3 island (−3.6 V, 0.01 nA). (**f_3_**) DFT-calculated STM images (−3.6 V, 0.01 nA) with overlaid model [57].

Chen et al. [27] synthesized oligophenylene macrocycles on an Ag(111) surface via a solvent-free C–C coupling reaction using 4,4⁗-dibromo-meta-quinquephenyl (DMQP) molecules, designating them as (n)-honeycombene (where n represents the number of phenylene units). The reaction forming (30)-honeycombene (composed of 30 benzene rings) is illustrated in Figure 4a, where six DMQP molecules undergo cyclization via a debrominative coupling reaction. Figure 4(a_1_) shows an STM image of DMQP precursors adsorbed on Ag(111) at 260 K, revealing a long-range ordered supramolecular network in which molecules are connected via quadruple nodes. These nodes are likely formed by a combination of Br···H hydrogen bonds and Br···Br halogen bonds [55,56]. Upon heating to 640 K, the C–Br bonds cleaved and C–C bonds formed, leading to the successful synthesis of the (30)-honeycombene macrocycle (Figure 4(a_2_)). With a diameter of 4.0 nm, this is the largest shape-persistent, fully conjugated, and unsubstituted macrocycle reported to date. The (30)-HC macrocycles self-assemble on the Ag(111) surface, forming islands with hexagonal symmetry, as shown in Figure 4(a_3_). The hexagonal unit cell vectors have a length of 4.5 nm and an angle of 55°. Distinct gaps exist between the edges of two adjacent molecules, indicating that no covalent bonds have been formed between the macrocycles, indicating that the self-assembled structure is driven by van der Waals forces. Figure 4(a_4_) shows the differential conductance (dI/dV) mapping of the valence electronic structure in the macrocycle. As the bias voltage increased, a pronounced localization of the density of states (DOS) at the corner edges of the macrocycle was observed. The observed features can be qualitatively explained by large-wavelength amplitude modulations within the delocalized π-type molecular orbitals of the conjugated ring.

Figure 4b shows the macrocycles synthesized after annealing DMTP molecules on Ag(111) at 640 K. An STM image of a single macrocycle is shown in Figure 4(b_1_). Compared to (30)-honeycombene, (18)-honeycombene forms hexagonal islands (Figure 4(b_2_)) where two red protrusions appear between the edges of adjacent molecules, possibly corresponding to Br atoms. Its electronic structure resembles that of (30)-honeycombene: increasing the bias voltage shifts the DOS localization from the edges to the corners (Figure 4(b_4_)). The behaviors of single isolated molecules and molecules within the islands are similar (Figure 4(b_5_)), but the maxima for the latter occur at higher bias voltages, as indicated by the comparison of the dI/dV spectra in Figure 4(b_3_).

In addition to regular hexagonal honeycombs, annealing the DMQP precursor on Ag(111) also yields three byproducts: a square “(20)-HC”, a pentagonal “(25)-HC”, and a heptagonal “(35)-HC” strained ring, as shown in Figure 4c. Figure 4(c_1_) shows the formation of the icosaphenylene square ring after the reaction of four DMQP molecules, with the edges curved outward and exhibiting convex deformation. In contrast, the heptagonal cyclopentatriacontaphenylene macrocycle in Figure 4(c_3_) displays concave deformation. The pentagonal cyclopentacosaphenylene macrocycle (25 phenylenes, Figure 4(c_2_)) shows no significant deformation in the STM images. This strain arises partially from adsorption, which forces the macrocycle to adopt a planar geometry. The dI/dV map of the pentagon (Figure 4(c_4_)) reveals an enhanced local density of states (LDOS) at the edges for low bias voltages, with increasing localization of the LDOS at the corners as the bias voltage increases, once again indicating the conjugation of the ring. A qualitatively similar bias-dependent evolution of the LDOS was found for the heptagon (Figure 4(c_5_)).

Fan et al. [57] also investigated the cyclization behavior of DMTP precursor molecules using Cu(111) as a substrate. The substrate temperature was set to 300 K and 550 K during DMTP deposition onto the Cu(111) surface to study the structural configurations, as outlined in Figure 4d. At 300 K, the molecules coordinated with Cu to form one-dimensional polymers, appearing as elongated islands composed of zigzag chains (Figure 4(e,e_2_)). The lattice constant along the chain direction was 26.5 Å (Figure 4(e_1_)), consistent with DFT calculations. When the substrate deposition temperature was increased to 440 K (Figure 4(e_3_)), uniform islands of 1D coordination polymers were formed, but no evidence of C–C bond formation was observed. Upon further increasing the substrate deposition temperature to 550 K (Figure 4f), distinct structures emerged. An ordered array of hexagonal rings, each with a diameter of 21.3 Å, was obtained. Each hexagon consisted of six DMTP molecules (one at each vertex), encompassing 18 phenylene units. This ring can be termed octadecaphenylene or, alternatively, superbenzene. The unit cell dimensions of the hexagonal lattice are shown in Figure 4(f_2_). Comparing the unit cell sizes and combining that with the DFT calculation results (Figure 4(f_3_)), it is indicated that the superbenzene rings are connected by covalent C–C bonds rather than C–Cu–C coordination bonds. Driven by van der Waals interactions, the superbenzene molecules aggregate into an ordered array with a hexagonal unit cell. Due to the large diameter of the superbenzene molecules, the nanoscale cavities could potentially encapsulate various quantum dots, such as metal and semiconductor nanoparticles or large organic molecules.

### 3.2. Post-Coordination C–C Coupling

Halogenated hydrocarbons are widely used precursors in on-surface synthesis for forming 2D metal–organic frameworks (MOFs) and covalent organic frameworks (COFs) [58,59,60,61]. Assisted by the metal surface, C–X (X: Cl, Br, I) bond cleavage occurs, and rationally designed halogenated molecules can yield diverse topological structures [62,63].

**Figure 5:**
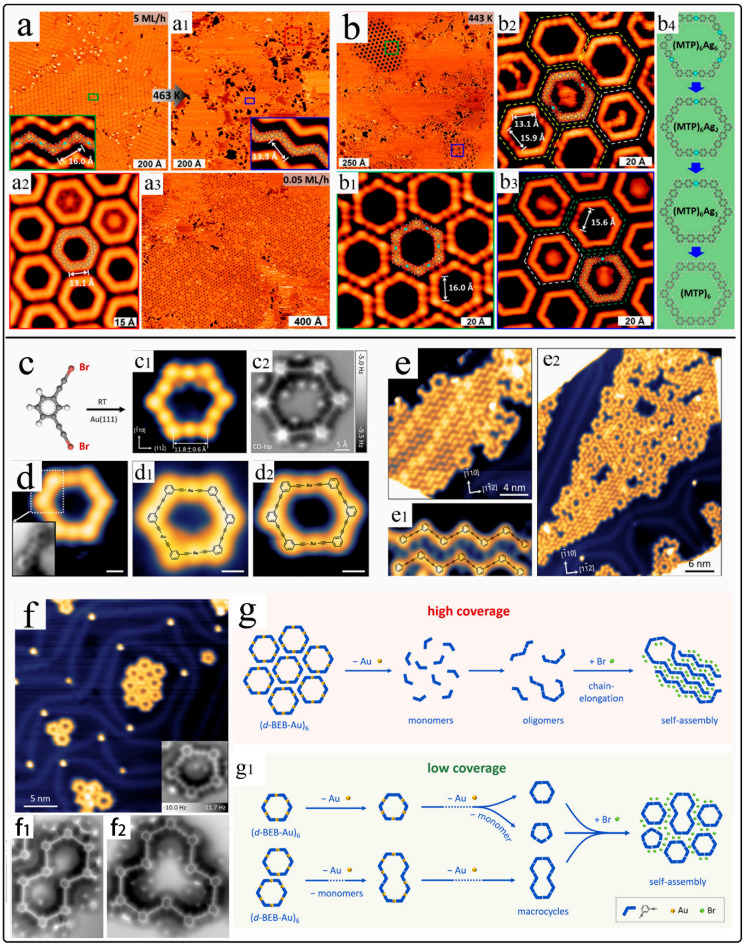
(**a**) DMTP/Ag(111) at 353 K (0.6 ML, 5 ML/h). The inset shows a magnified view (1.4 V, 0.08 nA). (**a_1_**) After annealing the sample to 463 K. The inset shows the blue framed region (1.5 V, 0.13 nA). (**a_2_**) Zoomed in STM image of the red framed region (1.5 V, 0.13 nA). (**a_3_**) DMTP/Ag(111) at 463 K (0.5 ML, 0.05 ML/h) (0.29 V, 0.19 nA). (**b**) DMTP/Ag(111) at 443 K (0.5 ML, 0.5 ML/h); (**b_1_**) Zoomed in image of the green framed region in panel b (1.4 V, 0.15 nA). (**b_2_**) Partially metalated hyperbenzene (0.44 V, 0.19 nA). (**b_3_**) Blue framed region in panel b (0.88 V, 0.17 nA). (**b_4_**) Scheme for the evolution of cyclic (MTP-Ag)_6_ to (MTP)_6_ [19]. (**c**) Molecular structure of BEB. (**c_1_**) STM of a cyclic (d-BEB-Au)_6_ (−700 mV, 20 pA). (**c_2_**) nc-AFM image of a (d-BEB-Au)6 intermediate using a CO-functionalized tip (“d-BEB” represents the dehalogenated BEB). (**d**–**d_2_**) STM images of three partially demetallized organometallic rings. (**e**) STM image of graphdiyne zigzag chains. (**e_1_**) Zigzag chains with molecular models. (**e_2_**) Overview STM images of coexisting chains and macrocycles (−700 mV, 20 pA). (**f**) Large-scale STM image of graphdiyne macrocycles (−700 mV, 20 pA). The inset is pentagonal macrocycle. (**f_1_**,**f_2_**) nc-AFM images of 10- and 12-membered macrocycles, (2.94 × 3.85, 3.85 × 3.85 nm^2^, respectively). (**g**) High-coverage and (**g_1_**) low-coverage intermediates generate graphdiyne chains and graphdiyne macrocycles, respectively [66].

Using solvent-free on-surface synthesis with DMTP as the precursor molecule, Fan et al. [19] illustrated the mechanism governing the competition between cyclization and chain growth under pseudo-high-dilution conditions: cyclization (ring closure) is a first-order reaction, while chain growth through the attachment of another precursor is a second-order reaction. With the concentration decreases, first-order cyclization competes increasingly successfully against second-order chain extension. Their work involved a series of comparative experiments. First, they studied high-concentration conditions: depositing 0.6 monolayers (ML) of DMTP molecules onto an Ag(111) surface at 353 K with a high flux (5 ML/h) resulted in islands of zigzag chains (Figure 5a). The superimposed model (inset) confirms that m-terphenyl (MTP) units occupy the corners with Ag atoms (bright dots) at the centers, demonstrating the formation of C–Ag–C bonds between MTP units. After annealing the sample to 463 K (Figure 5(a_1_)), metal atoms detached at this temperature, and C–C coupling formed zigzag oligophenylene chains and hexagonal biphenylene rings (as shown by the overlaid molecular models in Figure 5(a_1_,a_2_)). Statistical analysis revealed a yield of 94% for zigzag oligophenylene chains and 6% for biphenylene rings after annealing. The concentration of 0.6 ML (2D) corresponds to approximately 0.6 mol/L (3D), which is significantly higher than the precursor concentrations (<10^−4^ mol/L) typically used in high-dilution cyclization reactions [64,65]. To achieve pseudo-high-dilution conditions, the researchers deposited DMTP onto Ag(111) at 463 K with a flux f = 0.05 ML/h. Under these conditions, the yield of the biphenylene macrocycle dramatically increased to 84% (Figure 5(a_3_)).

To elucidate the cyclization mechanism during the organometallic intermediate stage in the formation of cyclopoly (p-phenylene) macrocycles, Fan et al. conducted an investigation of the reaction at 443 K under consistent flux conditions, as shown in Figure 5b. The hexagon with a larger cavity (Figure 5(b_1_)) was identified, based on its side length and the overlaid molecular model, as the organometallic (MTP–Ag)_6_ macrocycle, comprising six MTP units and six Ag atoms. Additionally, hexagonal rings of (MTP)_6_Ag_x_ (x < 6) were observed. Figure 5(b_2_) shows STM images of (MTP)_6_ and the doubly metalated (MTP)_6_Ag_2_. The white dashed outline shows the superbenzene (MTP)_6_, whose six sides are equal in length and identical to those in Figure 5(a_2_). The (MTP)_6_Ag_2_ appears as an elongated hexagon with four short sides and two long sides. The measurements and analysis indicate that the four short sides connect MTP units via C–C bonds, while the two long sides connect MTP units via C–Ag–C bonds. A hexagon (MTP)_6_Ag with only an Ag atom, featuring one long side and five short sides, is observed in Figure 5(b_3_). This hexagon, (MTP)_6_Ag, represents the final organometallic intermediate. The elimination of the remaining Ag atom ultimately forms the cyclopoly (p-phenylene). Based on these observations, the authors propose that macrocycle formation occurs during the organometallic phase under pseudo-high-dilution conditions. This reaction mechanism initially leads to the formation of the organometallic (MTP–Ag)_6_ macrocycle, which then evolves into the final cyclopoly (p-phenylene) through the stepwise elimination of Ag atoms, as depicted in the reaction scheme in Figure 5(b_4_). This study provides important guidance for the high-yield on-surface synthesis of macrocycles.

Liu et al. [66] conducted a comprehensive study on the dehalogenation and coupling reactions of 1,3-bis(2-bromoethynyl)benzene (BEB) on Au(111), revealing a series of reaction steps and the transformation of various chemical species. The molecular structure of BEB is shown in Figure 5c. After depositing BEB on Au(111) at RT, a uniform hexagonal structure with a side length of 11.8 ± 0.6 Å was observed, as shown in Figure 5(c_1_). The corresponding nc-AFM image (Figure 5(c_2_)) shows phenylene units at the vertices of the hexagon. The bright spots between the benzenes (Figure 5(c_1_)) are attributed to Au adatoms, which play a crucial role in the organometallic intermediate, designated as the (d-BEB–Au)_6_ ring. Annealing the sample at 323 K led to a structural distortion (Figure 5(d–d_2_)), with the disappearance of bright spots corresponding to Au adatoms on one side of the hexagon and a reduction in side length. The inset in Figure 5d shows two additional bright spots between adjacent phenylene units, indicative of conjugated C≡C triple bonds, suggesting the formation of butadiyne linkages post-demetallation. Increasing the annealing temperature to 353 K resulted in the emergence of a mixture of zigzag chains and macrocycles, as shown in Figure 5(e,e_2_). The distance between two phenylene units in the zigzag chains is 9.1 ± 0.2 Å, identical to the side length of the macrocycle after demetallation. Structural analysis, corroborated by the molecular overlay model presented in Figure 5(e_1_), unequivocally identifies the zigzag chains as covalent graphdiyne chains. Although both covalent chains and rings form after complete demetallation, the ratio of graphdiyne macrocycles to chains is highly sensitive to the initial surface coverage of the (d-BEB–Au)_6_ intermediate. As the coverage of the (d-BEB–Au)_6_ rings decreases, the yield of macrocycles increases significantly. When the coverage of (d-BEB–Au)_6_ rings was reduced to approximately 4.8 × 10^12^ molecules/cm^2^, a macrocycle yield of up to 95% was achieved (Figure 5f). In addition to hexagonal macrocycles, other cyclic structures were observed, including pentagonal rings (inset in Figure 5f) as well as 10-membered (Figure 5(f_1_)) and 12-membered macrocycles (Figure 5(f_2_)).

The authors investigated the competition mechanism between chain and ring formations under different surface coverages. The formation of uniform (d-BEB–Au)_6_ hexagonal intermediates after dehalogenation is a thermodynamically controlled process. The reversible nature of organometallic bonds enables the transformation of metastable species into the global minimum energy product [67,68]. In contrast, the coupling of terminal alkynes following the demetallation of (d-BEB–Au) is a kinetically controlled process, as the formed covalent bonds are irreversible at the annealing temperature. At higher surface coverage of the (d-BEB–Au)_6_ intermediate (Figure 5g), d-BEB monomers generated by thermal demetallation extend into open-chain oligomers, with ring formation competing with chain elongation. The rapid elongation of these oligomers can exceed the length required for macrocyclization, analogous to the conditions observed in solution [69]. Although the conversion from linear oligomers to cyclic products is thermodynamically favorable, inappropriate chain lengths and inter-chain interactions hinder macrocycle formation, resulting in arrays of graphdiyne chains. On the other hand, at lower coverage of the (d-BEB–Au)_6_ intermediate (Figure 5(g_1_)), approaching high-dilution conditions [70], stepwise demetallation under mild annealing temperatures allows individual (d-BEB–Au)_6_ hexagons to directly transform into covalent graphdiyne macrocycles (upper part of Figure 5(g_1_)). For small aggregates of a few (d-BEB–Au)_6_ units, the templating effect of the Au(111) surface also promotes macrocycle formation through the merging of closely situated intermediate rings and intramolecular cyclization (lower part of Figure 5(g_1_)). The mechanical flexibility of the butadiyne moieties aids these processes. This study highlights the intricate balance between thermodynamic and kinetic factors that govern the formation of organometallic and covalently bonded structures in on-surface synthesis.

## 4. Novel C–M–X Bonds

**Figure 6:**
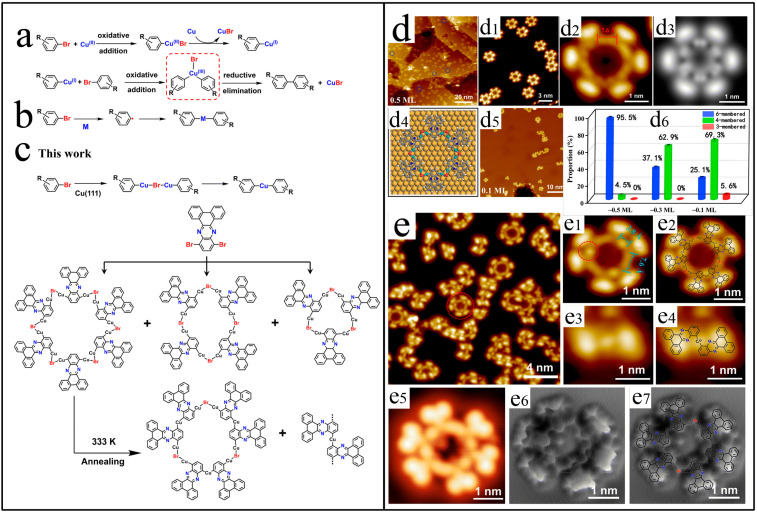
Schematic illustration of the Ullmann coupling reactions. (**a**) Possible mechanism of Cu-catalyzed Ullmann coupling in-solution chemistry. (**b**) The conventional reaction pathway of on-surface Ullmann coupling. (**c**) Reaction pathway of DBP-Br precursor on Cu(111). (**d**) Large-scale STM image recorded after depositing DBP–Br with a coverage of 0.5 ML on Cu(111) held at 300 K. (**d_1_**) Typical STM image showing uniform 6-membered nanorings. (**d_2_**) Close-up STM image of a single 6-membered nanoring. (**d_3_**) Simulated STM image of the 6-membered nanoring using a bias voltage of 500 mV. (**d_4_**) DFT-optimized molecular model of the 6-membered nanoring linked by C–Cu–Br–Cu–C bonds on the Cu(111) surface. (**d_5_**) Large-scale STM image recorded after depositing DBP–Br with a coverage of 0.1 ML on Cu(111) held at 300 K. (**d_6_**) Statistical analysis of the observed nanorings with different coverages on Cu(111). Scanning parameters: (**d**–**d_2_**,**d_5_**) V = 500 mV, I = 50 pA. (**e**) STM image of the sample annealed at 333 K. (**e_1_**) Zoomed in STM image of a 6-membered ring containing both C–Cu–Br–Cu–C and C–Cu–C bonds. The corresponding chemical structure is overlapped in (**e_2_**). (**e_3_**) Zoomed in STM image of a trans-dimer. The corresponding chemical structure is overlapped in (**e_4_**). (**e_5_**) Representative STM image and (**e_6_**) nc-AFM image of a 6-membered ring containing both C–Cu–Br–Cu–C and C–Cu–C bonds. The corresponding chemical structure is overlapped in (**e_7_**). Scanning parameters: V = 500 mV, I = 50 pA [28].

The Ullmann coupling reaction stands as one of the most important organic reactions for forming aryl–aryl bonds, holding significant importance in medicinal chemistry, natural product synthesis, and the preparation of optoelectronic materials [71,72]. Despite the advancements in achieving high reaction efficiency and selectivity over the past few decades, the reaction mechanism of Ullmann coupling remains elusive, primarily due to the difficulties in capturing and accurately characterizing reactive intermediates. Consequently, interpretations of the Ullmann coupling mechanism predominantly rely on theoretical calculations. For classical metal (M)-catalyzed Ullmann reaction systems, two prominent mechanisms are the M(I)–M(III) ionic mechanism (Figure 6a) and the single-electron transfer mechanism (Figure 6b) [73]. The precise reaction pathway for surface-assisted Ullmann coupling is still unknown [74]. The conventionally captured and characterized intermediates are C–M–C species (M = Au, Ag, Cu; Figure 6b) [27,74,75,76], which transform into covalent C–C bonds upon further activation. However, the process from the initial C–X (X = Cl, Br, I) dissociation to the formation of the C–M–C species remains unclear [77,78]. This ambiguity may be attributed to the short-lived nature of relevant C–M–X or similar intermediates, implying a negligible energy barrier for the conversion from C–M–X to C–M–C species.

In the context of solution-phase Ullmann coupling, the introduction of N-containing ligands enables the coupling reaction to proceed under mild conditions [79,80,81]. Inspired by this, Zhao et al. [28] investigated the reaction mechanism of N-doped brominated molecules on a Cu(111) surface using 10,13-Dibromodibenzo[a,c]phenazine (DBP-Br) as the precursor by monitoring the formation of organometallic intermediates (Figure 6c). Depositing DBP–Br molecules at approximately 0.5 monolayers (ML) onto Cu(111) at RT revealed large-scale six-membered rings (Figure 6d). Magnified STM images (Figure 6(d_1_,d_2_)) show that the six-membered ring consists of six DBP units, with an inter-unit distance of 7.6 Å (Figure 6(d_2_)), significantly longer than the typical C–Cu–C bond distance (3.8 Å) [26,82]. Two distinct spots are resolved within this linkage. Inspired by the C–Cu–Br bridging intermediates observed in solution-phase Ullmann coupling [79,81], this linkage was assigned to a C–Cu–Br–Cu–C motif, a view corroborated by DFT-simulated STM images (Figure 6(d_4_)). As the coverage decreased, the dominant nanoring product on Cu(111) at RT evolves from larger six-membered rings to smaller four-membered and three-membered rings (Figure 6(d_5_)). These nanorings exhibit high thermal stability. This organometallic nanoring, connected via the novel C–Cu–Br–Cu–C bond, expands the family of functional organic nanorings.

Annealing the sample at 333 K for 20 min (Figure 6e) caused most six-membered nanorings to open and undergo morphological changes, as shown in Figure 6(e_1_,e_2_). Three linkages retain the C–Cu–Br–Cu–C connecting configuration, but the other three linkages shorten to 3.8 Å, corresponding to conventional C–Cu–C bonds in Ullmann coupling on Cu(111)51, where the bright spots correspond to Cu adatoms. Trans-dimers connected via C–Cu–C bonds were also observed after post-annealing at 333 K (Figure 6(e_3_,e_4_)). nc-AFM images (Figure 6(e_5_)) further confirmed the structures of organometallic intermediates connected by both C–Cu–Br–Cu–C and C–Cu–C bonds. This specific six-membered ring contains four C–Cu–C bonds and two C–Cu–Br–Cu–C bonds (Figure 6(e_6_,e_7_)). The C–Cu–Br–Cu–C species completely transformed into C–Cu–C linked chains at 353 K [28]. Based on the coexistence of C–Cu–Br–Cu–C and C–Cu–C bonded organometallic intermediates during the annealing-induced phase transition, the authors identified the C–Cu–Br–Cu–C linkage as the precursor state to the C–Cu–C bond, thereby filling the gap in the mechanism between C–X dissociation and C–Cu–C formation in surface-assisted Ullmann coupling reactions.

This C–Cu–Br–Cu–C organometallic intermediate was reported for the first time in on-surface synthesis. While introducing N atoms into the precursor molecule may be an indispensable factor for forming the C–Cu–Br–Cu–C intermediate, it is not the sole decisive parameter, as this intermediate has never been observed in the on-surface synthesis of other N-doped aryl halides [83,84,85]. Further research can focus on how the precursor structure promotes the formation of C–Cu–Br–Cu–C bonded intermediates by tuning parameters such as the nitrogen substitution position, the length and shape of the molecular backbone, the degree of charge transfer [86], and ring strain. This study pioneers new avenues for re-examining the reaction mechanisms of Ullmann coupling, both on surfaces and in solutions.

## 5. Hydrogen and Halogen Bonding

Hydrogen bonding, halogen bonding (XB), van der Waals forces, and π–π stacking have been extensively employed to construct numerous highly ordered supramolecular structures. Hydrogen bonding and XB are significant non-covalent interactions. The systems based on these interactions can avoid structural irreversibility in covalent reactions, enabling molecules to self-assemble into large-scale, defect-free domains, and they have been widely studied using crystallography and theoretical methods [87,88].

**Figure 7:**
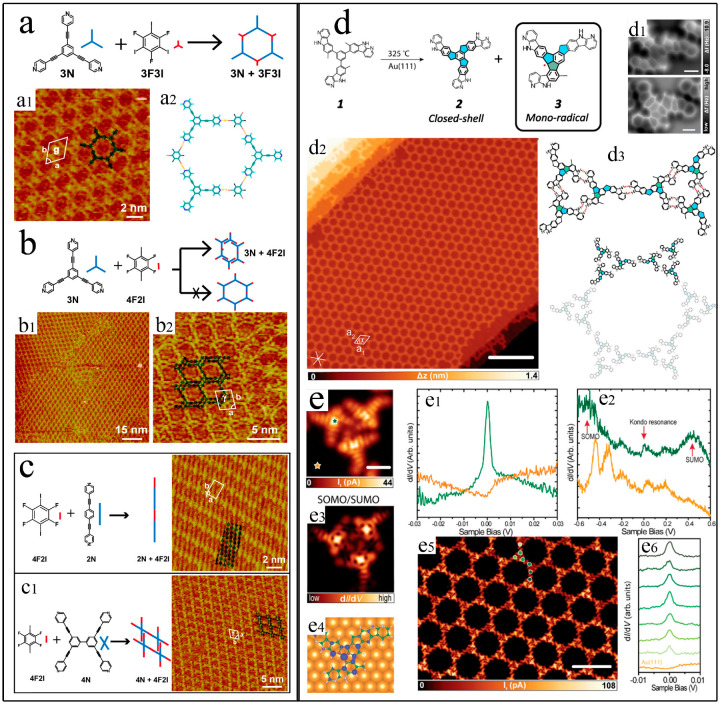
(**a**) Self-assembly of 3N and 3F3I results in the formation of a 3N/3F3I honeycomb structure. (**a_1_**) High-resolution STM image of the 3N/3F3I honeycomb structure on graphite (V = 888 mV, I = 1060 pA). (**a_2_**) Structural model of the 3N/3F3I honeycomb structure. (**b**) Schematic of the 3N/4F2I self-assembled porous structure. (**b_1_**) Large-scale STM image of the 3N/4F2I porous structure (V = 679 mV, I = 450 pA). (**b_2_**) High-resolution STM image of the 3N/4F2I porous structure (V = 725 mV, I = 545 pA). (**c**) High-resolution STM image of the 2N/4F2I linear structure (V = 758 mV, I = 513 pA). (**c_1_**) High-resolution STM image of the 4N/4F2I porous structure (V = 766 mV, I = 452 pA) [29]. (**d**) Synthetic route toward the formation of Products 2 and 3 on Au(111) after annealing the sample at 325 °C. (**d_1_**) High-resolution nc-AFM image of 3 (V = 1 mV, scale bar: 4 Å) and simulated nc-AFM image of Product 3 on Au(111) (scale bar: 4 Å). (**d_2_**) Constant-current overview STM image of the 2D-HBORF Kagome-honeycomb phase formed by Product 3 (V = 100 mV, I = 20 pA, scale bar: 31 nm). (**d_3_**) Molecular representation of the 2D-HBORF formed by Product 3. The hydrogen bonds are represented as red dashed lines. (**e**) Constant-height STM image of 3 at a low bias voltage (V = 1 mV, scale bar: 1 nm). (**e_1_**) Low-energy and (**e_2_**) medium-energy dI/dV spectra acquired on 3. Colored stars in (**e**) indicate the locations of the spectra. (**e_3_**) Simulated dI/dV map of the SOMO/SUMO. (**e_4_**) DFT-calculated spin density. (**e_5_**) Overview of the constant-height STM image of the Kagome-honeycomb phase (V = 5 mV, scale bar: 8 nm). (**e_6_**) Short-range dI/dV spectra acquired over a series of seven adjacent molecules [30].

Zheng et al. [29] investigated the formation of cyclic molecules and self-assembled supramolecular structures via different non-covalent bonds (hydrogen and halogen bonding) on a graphite surface by designing various ethynylpyridines (2N, 3N, 4N) and aryl halides (3F3I, 4F2I). The formation of a highly ordered 2D honeycomb network, achieved through the application of controlled electrical pulses (3.6 V) during scanning probe manipulation (Figure 7(a_1_)), demonstrates a potent strategy for directing the assembly of 3F3I and 3N molecules on surfaces. Structural modeling (Figure 7(a_2_)) reveals the underlying molecular recognition motif driving this assembly: each iodine atom at the meta position of a 3F3I molecule engages in a directional XB with an adjacent XB-accepting nitrogen site on a 3N molecule (yellow dashed lines, Figure 7(a_2_)). This specific, cooperative network of XB interactions provides the essential supramolecular synthons for the observed long-range periodic order and stability of the honeycomb architecture. The reaction pattern of the 3N/4F2I dyad is shown in Figure 7b. The application of several electrical pulses (3.6–4.2 V) yielded a complex porous structure (Figure 7(b_1_)). Figure 7(b_2_) shows a high-resolution STM image. The honeycomb-like network has a pore diameter of 4.8 nm and unit cell parameters of a = 3.4 ± 0.2 nm, b = 3.6 ± 0.2 nm, and γ = 79 ± 2°. Two antiparallel branches exist along the a-direction. XB bonds form between the iodine atoms of 4F2I and the pyridyl groups of 3N molecules on parallel lines, while hydrogen bonds form between the fluorine atoms of 4F2I and the hydrogen atoms of 3N on two antiparallel lines. The 3N/4F2I porous structure was stabilized and interconnected through hydrogen and XB bonds. Using the simple ditopic 4F2I as the XB donor and 2N and 4N molecules as XB acceptors, Figure 7c shows 2N molecules interacting with 4F2I via two pyridyl groups to form an XB-based binary linear structure. Figure 7(c_1_) shows the formation of an XB-based porous structure from 4N/4F2I. This structure was stabilized by a combination of hydrogen bonding and XB, similar to that observed for the 3N/4F2I binary XB-based porous structure. The authors pioneered a method for fabricating binary XB-based supramolecular structures, enabling the creation of XB-based binary open networks on surfaces and providing a new strategy for the future development of XB-based materials.

Under ultra-high vacuum (UHV) conditions, Frezza et al. [30] sublimated 1,3,5-tris(7-methyl-α-carbolin-6-yl)benzene (Precursor 1) onto an Au(111) surface. A temperature of 325 °C annealing triggered oxidative ring closure and cyclodehydrogenation, yielding Products 2–3 (Figure 7d). Product 2 accounted for approximately 40%, and Product 3 accounted for 60%. The structure of Product 3 was characterized using non-contact atomic force microscopy (nc-AFM) (Figure 7(d_1_)). The molecular structure of Product 3 features a fluoradene-derived central core covalently linked with 7-azaindole moieties. These trimers self-organize into dodecameric macrocycles (Figure 7(d_3_)), generating a Kagome-honeycomb phase with nanoscale pores 5.00 nm in diameter. The unit cell parameters are a_1_ = a_2_ = 6.40 nm and α = 60°. The hydrogen bonds in Product 3 favor the formation of the Kagome-honeycomb phase. This phase extended over hundreds of nanometers without defects, indicating the high quality and scalability of this supramolecular system.

To investigate the magnetic properties of the sample, STM/STS measurements were conducted at low bias voltages. STM imaging of Product 3 with a CO-modified tip in Figure 7e exhibits distinct bright lobes. The corresponding STS spectra show two key features: (i) a Kondo resonance at the Fermi level (Figure 7(e_1_)) and (ii) symmetric electronic states at ±0.5 eV, corresponding to the singly occupied molecular orbital (SOMO) and singly unoccupied molecular orbital (SUMO) (Figure 7(e_2_)). Spin-unrestricted DFT calculations predicted the presence of unpaired electrons in the SOMO orbital. The simulated SOMO/SUMO orbital maps (Figure 7(e_3_)) were consistent with the constant-height STM image. Figure 7(e_4_) displays the spin density of the lone electron in Product 3 on Au(111). The authors conclude that Product 3 possesses a paramagnetic moment S = 1/2, which is screened by the free electrons in the metal, giving rise to the Kondo effect. No spin excitation signals were found in the dI/dV maps, ruling out significant spin–spin interactions between adjacent molecules. The authors investigated magnetism over larger areas to verify the long-range magnetic order. STM images of the Kagome-honeycomb phase at different heights were taken at low bias voltages. Figure 7(e_5_) demonstrates that the magnetism of Product 3 extends throughout the supramolecular assembly. Figure 7(e_6_) shows the dI/dV spectra of seven adjacent molecules within Figure 7(e_5_), all of which exhibit characteristic Kondo resonances. This indicates that all molecules within the extensive self-assembly fit single-radical characterization. By designing and synthesizing a trifurcated precursor, the authors created a radical hydrogen-bonded supramolecular framework that was adsorbed onto a gold surface. This novel poly-π polyaromatic system with a fluorene core provides an organic radical with a stable S = 1/2 spin state on Au(111). This study represents a pioneering interdisciplinary effort that integrates supramolecular chemistry, π-magnetism, and on-surface synthesis, significantly advancing the field of supramolecular organic radical chemistry.

## 6. Conclusions

The field of on-surface-synthesized macrocycle chemistry is rapidly advancing, enabling the precise fabrication of structures ranging from simple cyclic assemblies to giant conjugated macrocycles, such as the 7 nm macrocycle. This progress is driven by strategies such as careful precursor molecular design and strict control of reaction conditions. Through the use of STM/nc-AFM and theoretical calculations, detailed structural and electronic characterizations of diverse macrocycles have been conducted, enhancing our understanding of macrocyclization processes and formation mechanisms. This review compiles recent developments in on-surface-synthesized macrocycles, focusing on their reaction pathways, cyclization mechanisms, and physicochemical properties as influenced by intermolecular interactions, analyzed from the perspective of bonding characteristics. We have detailed the reaction processes, intermediate formation, and stepwise evolution of different precursor molecules synthesizing macrocycles on solid surfaces under the effects of hydrogen bonding, halogen bonding, metal–ligand bonding, covalent bonding, and the newly discovered organometallic halogen bonding. The influence of external environmental factors, such as the substrate surface properties, annealing temperature, local electric field, molecular coverage, and deposition flux on the final macrocycle formation has been investigated. The subtle interplay between thermodynamic and kinetic factors governing macrocycle structure has been elucidated through the effects of molecular coverage and deposition flux. The mechanism governing the competition between cyclization and chain growth under high-dilution conditions has also been investigated. The discovery of the novel C–M–X bond has filled the mechanistic gap between C–X dissociation and C–Cu–C formation in Ullmann coupling. These studies involve the structural evolution process and microscopic reaction mechanisms of on-surface-synthesized macrocycles. These fundamental breakthroughs not only enrich the scope of surface synthesis but also pioneer new pathways for the preparation of functional nanostructures.

Looking ahead, research on on-surface-synthesized macrocycles is expected to advance towards functionalization, intelligent design, and systematization. On the one hand, researchers will place greater emphasis on the functional design of macrocycles, developing advanced molecular systems with specific optoelectronic, catalytic, or recognition properties. On the other hand, the integration of artificial intelligence and automation technologies will enhance efficiency and precision in on-surface synthesis research. Furthermore, the integration of on-surface-synthesized macrocycles with other nanostructures will emerge as a significant direction, paving the way for the construction of complex molecular machines and multifunctional nanodevices.
